# Are B waves of intracranial pressure suppressed by general anesthesia?

**DOI:** 10.1186/2045-8118-12-S1-O63

**Published:** 2015-09-18

**Authors:** Despina Afroditi Lalou, Joseph Donnelly, Marek Czosnyka, Eva Nabbanja, Matthew Garnett, John D Pickard, Zofia Helena Czosnyka

**Affiliations:** 1 Neurosurgery, University of Cambridge, UK

## Objective

Our previous study indicated that the magnitude of ICP slow vasogenic waves (also known as B waves), recorded during infusion study in hydrocephalic patients, was significantly suppressed when the study was performed under general anesthesia (GA). This was suggested to be secondary to decreased brain metabolism rate in patients under GA, as estimated by CSF production rate found lower than in conscious patients. Limitation of the previous study was that infusion test, limited in time (average 30 minutes), is not ideal for the detection of power of slow waves (up to 3 minutes in duration) using traditional spectral analysis. We continued research in this direction, comparing overnight ICP monitoring in hydrocephalus (conscious patients) with patients after TBI without intracranial hypertension (monitored under GA).

## Methods

Two groups of patients were studied: 30 with overnight ICP monitoring diagnosed for hydrocephalus and 30 consecutive TBI patients, without intracranial hypertension (confirmed by mean ICP< 18 mm Hg). The TBI patients were anesthetized and ventilated, with data recorded during first night of monitoring.

Mean ICP, pulse amplitude of ICP, magnitude of slow vasogenic waves (periods from 20 seconds to 3 minutes), and index of compensatory reserve RAP were compared using Mann-Whitney U test.

## Results

Overnight magnitude of slow waves was greater in conscious patients than in patients under GA (1.5+/-0.43 versus 0.7+/- 0.41 mm Hg; p<0.5*10-7). Compensatory index RAP was slightly lower (although insignificantly) in GA than in conscious patients (0.31+/-0.17 vs 0.4+/-0.18; p=0.066) indicating better compensatory reserve under GA . Pulse amplitude (peak to peak) was almost identical in two groups (5.7+/-4.1 mm Hg) and respiratory wave was greater in GA (ventilated) than in conscious patients (breathing spontaneously). Mean ICP was greater in GA than in conscious patients (13.8+/-2.9 vs 7.7+/-4.8 mm Hg; p=1*10-6).

## Conclusion

Results confirm previous observations that under GA magnitude of slow ICP waves tends to be lower than in conscious patients. This was observed in parallel with better compensatory reserve under GA, probably due to the fact that anesthetised patients had lower PaCO2 leading to a vasoconstriction. These differences should be taken into account in any methodology using the magnitude of slow ICP waves as a clinical biomarker.

